# Discovery of genomic loci associated with sleep apnea risk through multi-trait GWAS analysis with snoring

**DOI:** 10.1093/sleep/zsac308

**Published:** 2022-12-16

**Authors:** Adrian I Campos, Nathan Ingold, Yunru Huang, Brittany L Mitchell, Pik-Fang Kho, Xikun Han, Luis M García-Marín, Jue-Sheng Ong, 23andMe Research Team, Michelle Agee, Stella Aslibekyan, Adam Auton, Elizabeth Babalola, Robert K Bell, Jessica Bielenberg, Katarzyna Bryc, Emily Bullis, Briana Cameron, Daniella Coker, Devika Dhamija, Sayantan Das, Sarah L Elson, Teresa Filshtein, Kipper Fletez-Brant, Pierre Fontanillas, Will Freyman, Pooja M Gandhi, Karl Heilbron, Barry Hicks, David A Hinds, Karen E Huber, Ethan M Jewett, Yunxuan Jiang, Aaron Kleinman, Katelyn Kukar, Keng-Han Lin, Maya Lowe, Marie K Luff, Jennifer C McCreight, Matthew H McIntyre, Kimberly F McManus, Steven J Micheletti, Meghan E Moreno, Joanna L Mountain, Sahar V Mozaffari, Priyanka Nandakumar, Elizabeth S Noblin, Jared O’Connell, Aaron A Petrakovitz, G David Poznik, Anjali J Shastri, Janie F Shelton, Jingchunzi Shi, Suyash Shringarpure, Chao Tian, Vinh Tran, Joyce Y Tung, Xin Wang, Wei Wang, Catherine H Weldon, Peter Wilton, Matthew H Law, Jennifer S Yokoyama, Nicholas G Martin, Xianjun Dong, Gabriel Cuellar-Partida, Stuart MacGregor, Stella Aslibekyan, Miguel E Rentería

**Affiliations:** QIMR Berghofer Medical Research Institute, Brisbane, QLD, Australia; School of Biomedical Sciences, Faculty of Medicine, University of Queensland, Brisbane, QLD, Australia; Institute for Molecular Bioscience, University of Queensland, Brisbane, QLD, Australia; QIMR Berghofer Medical Research Institute, Brisbane, QLD, Australia; School of Biomedical Sciences, Faculty of Health, Queensland University of Technology, Brisbane, QLD, Australia; 23andMe, Inc., Sunnyvale, CA, USA; QIMR Berghofer Medical Research Institute, Brisbane, QLD, Australia; School of Biomedical Sciences, Faculty of Medicine, University of Queensland, Brisbane, QLD, Australia; Division of Cardiovascular Medicine, Department of Medicine, Stanford University School of Medicine, Stanford, CA, USA; Program in Genetic Epidemiology and Statistical Genetics, Harvard University T.H. Chan School of Public Health, Boston, MA, USA; QIMR Berghofer Medical Research Institute, Brisbane, QLD, Australia; School of Biomedical Sciences, Faculty of Medicine, University of Queensland, Brisbane, QLD, Australia; QIMR Berghofer Medical Research Institute, Brisbane, QLD, Australia; 23andMe, Inc., Sunnyvale, CA, USA; QIMR Berghofer Medical Research Institute, Brisbane, QLD, Australia; School of Biomedical Sciences, Faculty of Health, Queensland University of Technology, Brisbane, QLD, Australia; Memory and Aging Center, University of California, San Francisco, San Francisco, CA, USA; Weill Institute of Neurosciences, Department of Neurology, University of California, San Francisco, San Francisco, CA, USA; QIMR Berghofer Medical Research Institute, Brisbane, QLD, Australia; Genomics and Bioinformatics Hub, Brigham and Women’s Hospital, Boston, MA, USA; Department of Neurology, Brigham and Women’s Hospital and Harvard Medical School, Boston, MA, USA; 23andMe, Inc., Sunnyvale, CA, USA; QIMR Berghofer Medical Research Institute, Brisbane, QLD, Australia; 23andMe, Inc., Sunnyvale, CA, USA; QIMR Berghofer Medical Research Institute, Brisbane, QLD, Australia; School of Biomedical Sciences, Faculty of Medicine, University of Queensland, Brisbane, QLD, Australia; School of Biomedical Sciences, Faculty of Health, Queensland University of Technology, Brisbane, QLD, Australia

**Keywords:** sleep apnea, snoring, genetics, GWAS

## Abstract

**Study Objectives:**

Despite its association with severe health conditions, the etiology of sleep apnea (SA) remains understudied. This study sought to identify genetic variants robustly associated with SA risk.

**Methods:**

We performed a genome-wide association study (GWAS) meta-analysis of SA across five cohorts (*N*_Total_ = 523 366), followed by a multi-trait analysis of GWAS (multi-trait analysis of genome-wide association summary statistics [MTAG]) to boost power, leveraging the high genetic correlation between SA and snoring. We then adjusted our results for the genetic effects of body mass index (BMI) using multi-trait-based conditional and joint analysis (mtCOJO) and sought replication of lead hits in a large cohort of participants from 23andMe, Inc (*N*_Total_ = 1 477 352; *N*_cases_ = 175 522). We also explored genetic correlations with other complex traits and performed a phenome-wide screen for causally associated phenotypes using the latent causal variable method.

**Results:**

Our SA meta-analysis identified five independent variants with evidence of association beyond genome-wide significance. After adjustment for BMI, only one genome-wide significant variant was identified. MTAG analyses uncovered 49 significant independent loci associated with SA risk. Twenty-nine variants were replicated in the 23andMe GWAS adjusting for BMI. We observed genetic correlations with several complex traits, including multisite chronic pain, diabetes, eye disorders, high blood pressure, osteoarthritis, chronic obstructive pulmonary disease, and BMI-associated conditions.

**Conclusion:**

Our study uncovered multiple genetic loci associated with SA risk, thus increasing our understanding of the etiology of this condition and its relationship with other complex traits.

## Graphical Abstract

**Graphical Abstract:**
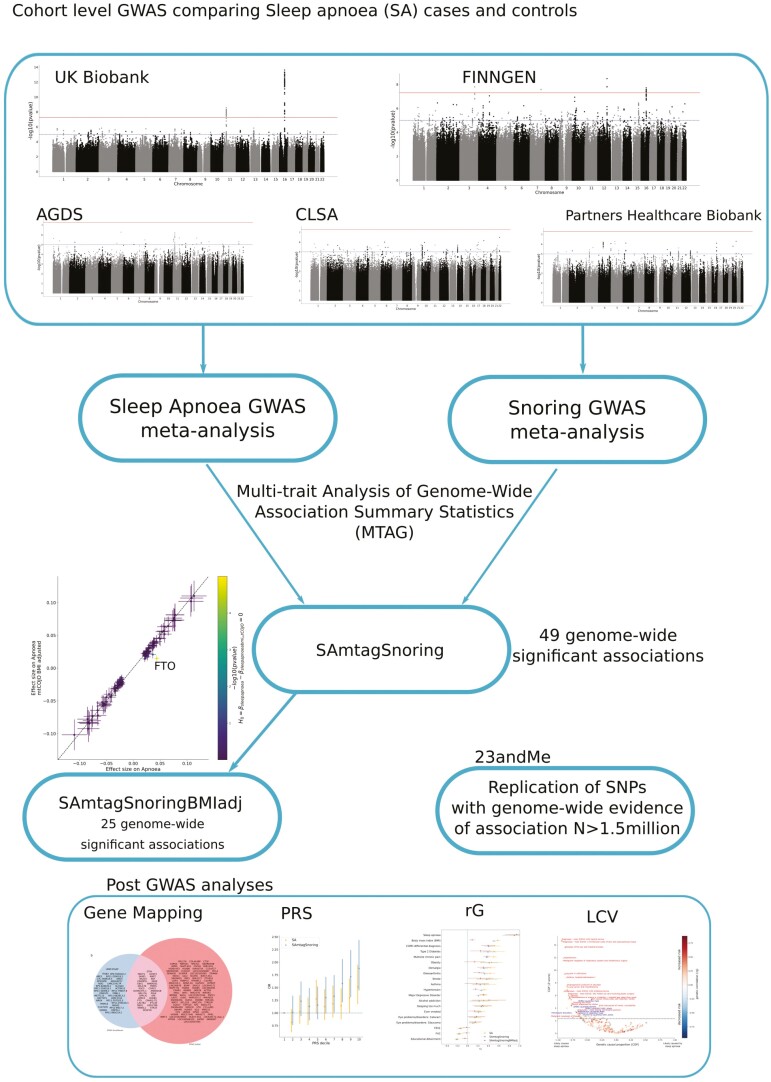

**Statement of Significance**

Sleep apnea (SA) is characterized by episodes of halted breathing during sleep. It is associated with an increased risk of hypertension, stroke, and increased levels of reactive oxygen species in the blood, which increase oxidative stress in the body. Between 25% and 75% of individual risk of presenting SA is heritable (i.e. explained by genetic differences). In this study, we combined SA and genetic data from large cohorts from Australia, Canada, Finland, the United States, and the United Kingdom to identify genetic variants associated with SA risk. We also explore the genetic relationship between SA and body mass index and other complex traits and diseases.

## Introduction

Sleep apnea (SA) is a disorder characterized by episodes of halted breathing during sleep, which leads to frequent arousal and intermittent hypoxia [1]. The most common type of SA is obstructive SA, which affects 9%–55% of adults and 1%–9.5% of children [2–5]. Obstructive SA is a complex disease with multiple underlying mechanisms and risk factors; these include craniofacial structure differences, decreased width of the upper airways, increased body mass index (BMI), or a reduced function of the pharyngeal dilator muscles, all of which contribute to the collapse of the upper airways and subsequent apneas and hypopneas [6–8].

SA is associated with several factors, including BMI, male sex, older age, craniofacial, or upper-airway abnormalities, smoking, alcohol consumption, cardiovascular disease, and family history of sleep apnea [9]. Furthermore, SA can lead to mental and physical fatigue, which is associated with an increase in the risk of motor accidents [10], and a decrease in mental well-being and overall quality of life [11]. In addition, SA has also been associated with an increased risk of hypertension [12], stroke [13], and increased levels of reactive oxygen species in blood, which increase oxidative stress in the body [14, 15].

Obesity (i.e. commonly determined as BMI > 30) is correlated with a higher SA risk [16]. In fact, one of the most important modifiable risk factors for SA is BMI. Obesity increases the risk for SA through the aggregation of fat deposits in the upper respiratory tract, which narrows the throat and induces a decrease in muscle activity, potentially leading to hypoxic and apneic episodes that lead to SA [17]. Therefore, it is important to consider the potential influences of BMI while studying SA.

The heritability of SA is estimated to be between 35% and 75% [18, 19], but familial aggregation seems to be partially independent of bodyweight [20], suggesting an independent germline component. Despite an estimated population prevalence of at least 5%, many SA cases go undiagnosed until other related diseases begin to display [21, 22]. Therefore, an increased understanding of the genetic architecture of SA could help generate risk prediction models, prompting earlier detection, and providing an important groundwork for the development of interventions and therapies. In addition, having information on the effect of genetic variants on SA risk could enable inference of its causal relationship with other conditions using methods such as Mendelian randomization [23]. Although some candidate gene studies for SA have yielded a few putatively associated genes [24, 25], genome-wide association studies (GWAS) have failed to replicate those associations [26–28]. In fact, GWAS have identified very few genome-wide significant loci robustly associated (i.e. with evidence of replication in an independent cohort) with SA to date.

SA is likely a highly polygenic trait, with many variants of small effect size contributing to the genetic liability of developing this condition. Thus, most studies with modest sample sizes will be underpowered to identify the majority of these risk variants and are susceptible to false-positive associations. Furthermore, the number of diagnosed cases of SA within existing large population cohorts is low. In a sample of 500 000 individuals, the expected number of SA cases (assuming a conservative prevalence of ~5%) would be ~25 000. However, in the UK Biobank (UKB) (~500 000 individuals), only ~8000 SA cases have been recorded. That is likely explained by the fact that SA is recognized as an underdiagnosed condition because those affected are unable to gain awareness about their condition or may confuse it with habitual snoring [21, 22]. Underdiagnosis further reduces power as many real cases may be labeled as unaffected controls in a standard analysis. Thus, combining large samples through meta-analysis and replicating findings in large, independent studies are essential steps to uncover reliable results.

Here, we conducted a GWAS meta-analysis of SA across five cohorts. Then, we employed multi-trait analysis of genome-wide association summary statistics (MTAG) to combine our results with a snoring GWAS meta-analysis across five cohorts to boost statistical power by leveraging the high genetic correlation between SA and snoring [29]. We also performed additional sensitivity analyses to control for the genetic effects of BMI and identify loci associated with SA independently from BMI. We sought to replicate lead single nucleotide polymorphisms (SNPs) in an independent sample from 23andMe, Inc. and further explored the genetic underpinnings of SA through gene-based tests and genetic correlation analyses. Finally, we constructed polygenic scores and predicted SA using a leave-one cohort-out (LOO) cross-validation framework. Our analyses can be interpreted as a proxy for obstructive SA, given its higher prevalence than central SA [3, 30].

## Methods

### Sample information and phenotype ascertainment

This study analyzed GWAS data from five cohorts from the United Kingdom (UKB), Canada (Canadian Longitudinal Study of Aging; CLSA) [31, 32], Australia (Australian Genetics of Depression Study; AGDS), the United States (Partners Healthcare Biobank), and Finland (FinnGen). The total sample size for each cohort, and the number of cases and controls are listed in Table 1. For each cohort, SA cases were defined using participant-reported diagnosis or ICD diagnostic codes available in electronic health records (ICD-9: 327.23 and ICD-10: G47.3). In CLSA and AGDS, SA was defined based on the answer to the item “Stop breathing during sleep” (see Supplementary Methods for individual cohort details). Self-reported snoring cases were excluded from the analyses for the SA GWAS across the UKB, CLSA, and AGDS cohorts. An overview of the analysis pipeline used for SA discovery analysis is available in the Supplementary Figure S1.

**Table 1.** Cohort and prevalence overview

Cohort	Total sample size	Apnea GWAS cases	Apnea GWAS controls	Snoring GWAS cases	Snoring GWAS controls
UK-Biobank	408 317	7902	248 112	152 303	256 014
Finngen	66 216	9096	57 120	4270	61 946
Partners Biobank	20 047	3102	16 945	4175	15 872
CLSA	18 427	3391	9615	6852	10 736
AGDS	10 359	1517	5838	4450	5907
Total	523 366	25 008	337 630	172 050	350 475

GWAS cases and controls correspond to participants passing quality control as described in the methods.

### GWAS

All GWA studies included the following covariates, namely, age, sex, batch (where relevant), and genetic ancestry principal components derived from genotype data. Standard quality control filters were applied at both the sample and variant levels. Variants were excluded from the analyses if they had a low minor allele frequency (MAF < 0.01) or low imputation quality score (INFO < 0.6). Individuals were excluded based on excess missingness, heterozygosity, or evidence of a deviation from European ancestry based on genetic principal components. For each cohort, a GWAS was performed using logistic regression models and including random effects to account for cryptic relatedness where relevant (Supplementary Methods). For the UKB snoring GWAS, we used the summary statistics from our previously published GWAS for snoring [33]. We obtained FinnGen GWAS results for SA and snoring from the open-access FinnGen resource (Freeze 3).

### GWAS meta-analyses

Sample-size weighted (*p*-value-based) meta-analyses for SA and snoring were performed (separately for each phenotype) across the five cohorts described above using METAL (v2020-05-05) [34]. Studies were weighted according to their effective sample size as described by the equation: *N*eff = 4/(1/*N*cases + 1/*N*controls), as recommended for studies with different levels of case-control imbalance (Supplementary Methods).

### Multi-trait GWAS analyses

We used MTAG to boost the statistical power for the discovery of SA-associated loci. MTAG performs a generalized meta-analysis of GWAS summary statistics for different but high genetically correlated traits while accounting for potential sample overlap [29]. For this study, we performed MTAG analyses combining our SA and snoring meta-analyses. That is possible given the high genetic correlation between these traits (*r*_*g*_ ~ 0.8) [33] and the observation that snoring is one of the primary symptoms of SA, the most common type of SA [7].

### BMI adjustment

Given the clear relationship between SA, snoring, and BMI, we performed a secondary analysis adjusting our GWAS results (both the meta-analysis and the MTAG) for the effect of BMI. To adjust for BMI while avoiding biases due to collider bias, (i.e. the emergence of a spurious association between a pair of variables when a common outcome is modeled as a covariate) [35], we used multi-trait-based conditional and joint analysis (mtCOJO) [36, 37]. As a sensitivity analysis, we also repeated the SA meta-analysis adjusting for BMI as a covariate. Including BMI as a covariate was only done for the AGDS, CLSA, and UK-Biobank cohorts. These results were compared to the unadjusted and mtCOJO-adjusted analyses using bivariate LD-score regression and by comparing the effect size of SNPs with suggestive evidence of association.

### 23andMe replication GWAS

We sought to replicate variants identified in the discovery phase in an independent sample of participants from the 23andMe cohort (*N* = 1 477 352). Cases were ascertained based on the question “Have you ever been diagnosed with, or treated for any of the following conditions?” with one of the choices being “Sleep apnea” (Yes = 175 522; No = 1 301 830). Methods and results from this GWAS have been presented at the 2018 American Society for Human Genetics annual conference [38]. Briefly, a logistic regression GWAS was performed using SA as the dependent variable while adjusting for sex, age, BMI, genetic principal components, and genotype array. Participants provided informed consent and participated in the research online, under a protocol approved by the external AAHRPP-accredited IRB, Ethical & Independent Review Services (E&I Review). Only unrelated participants of European ancestry who provided consent were included in the analysis.. We defined evidence of replication after correcting for the number of significant variants with data available for replication per GWAS analysis. That is *p* < .01 for the SA meta-analysis, *p* < .0016 for the SA plus snoring MTAG and *p* < .002 for the SA plus snoring MTAG adjusted for BMI.

### Gene-based association tests and eQTL colocalisation

We used the “set-based association analysis for human complex traits” fastBAT method, which performs a set-based enrichment analysis using GWAS summary statistics while accounting for linkage disequilibrium (LD) between SNPs [39]. Statistical significance was defined using the Bonferroni method for multiple testing correction (*p* < 2.07e−6). Genes identified as statistically significant were further assessed for expression quantitative trait loci (eQTL) colocalisation using the *COLOC* [40] package in R. Briefly, we integrated our GWAS summary data with *cis*-eQTL data from whole blood, esophagus, adipose, and lung tissue from GTEx V8 [41] to estimate the posterior probability that GWAS signals co-occur with eQTL signals while accounting for LD structure. This method estimates the posterior probabilities (PP) for five different scenarios. The scenario of interest is colocalisation due to associations with both traits through the same SNPs (PP4). A threshold of PP4 ≥ 0.8 was considered as evidence for colocalisation of GWAS signals and eQTL signals at the region of interest (Supplementary Methods).

### S-MultiXcan-based eQTL integration

Integration of eQTL with GWAS results interrogates whether the associations observed are consistent with changes in gene expression mediating the trait under study. This study integrated our GWAS results with eQTL data from GTEx using S-MultiXcan [42], as implemented in the Complex Traits Genetics Virtual Lab (CTG-VL). This method employs a multiple-regression of the phenotype on the predicted gene expression across multiple tissues based on eQTL data. When using only GWAS summary statistics, single-tissue associations are performed using S-PrediXcan, and joint effects from the single-tissue results are estimated using an approximation similar to that of the conditional and joint multiple-SNP analysis [43]. Contrary to the eQTL colocalisation described above, this analysis employs the whole GWAS summary statistics and is not restricted only to genes identified using fastBAT or other gene-based tests.

### Heritability and genetic correlations

We used LD score regression to estimate the SNP-based heritability (*h*_SNP_^2^) for the SA meta-analysis. Given that samples were not specifically ascertained for SA, we assumed the overall sample and population prevalence for SA to be the prevalence estimated across cohorts (0.05) that are consistent with reported epidemiological estimates [2]. Genetic correlations (*r*_*g*_) between SA and 1522 phenotypes (with available GWAS summary statistics) were estimated using bivariate LD score regression in CTG-VL [44] based on a common set of HapMap3 variants. The Benjamini–Hochberg false discovery rate (FDR) at 5% was used to define statistical significance.

### Polygenic risk scoring

To assess the external validity of the GWAS, we performed polygenic-based prediction on a target sample of 9221 unrelated Australian adults from the AGDS [45] with complete data. Briefly, the meta and MTAG analyses were repeated, leaving out the AGDS cohort to avoid sample overlap. We employed the SBayesR method to obtain the conditional effects of the studied variants, thus avoiding inflation due to correlated SNPs in LD [46]. SBayesR estimates the SNP multivariate effect sizes using GWAS summary statistics and SNP correlations using an LD-matrix. Here, we used the LD-matrix for 2.8M variants reported in Lloyd-Jones and Zeng et al. [27, 46], which is publicly available (10.5281/zenodo.3350914). SBayesR parameters included four mixture components (starting values = 0.95, 0.01, 0.02, 0.01) with default scaling factors (0, 0.01, 0.1, 1), chain length of 25 000, and burn-in of 5000. The SNP conditional effect sizes obtained from SBayesR were then used for polygenic scoring using HRCr1.1 imputed genotype dosage data in plink v1.9. Polygenic risk scoring (PRS) were calculated by multiplying the effect size of a given risk allele (obtained from the GWAS summary statistics) by the imputed number of risk alleles (using dosage probabilities) present in each individual. SNP scores were then summed across all loci. The association between PRS and SA in AGDS was assessed using a logistic regression model (python *statsmodels*). SA_PRS_ was the predictive variable of interest, with age, sex, and the first 10 genetic principal components included as covariates in Nagelkerke’s pseudo *R*^2^. Finally, binary classifiers based on logistic regression were built, including age and sex (base model) or age, sex, and the PRS of interest (SA_PRS_ or SAmtagSnoring_PRS_). These classifiers were used to assess the polygenic predictive ability further. The sample was divided randomly into training and testing datasets of equal sizes. Then, the classifier’s ability to predict SA was assessed using the area under the receiver operating characteristic (ROC) curve. To avoid potential biases from the random division of training and testing datasets, the procedure was repeated 100 times to estimate a mean area under the curve (Supplementary Methods).

### Latent causal variable analysis

The latent causal variable (LCV) method leverages GWAS summary statistics to estimate whether a causal association can explain a genetic correlation between traits rather than horizontal pleiotropy (i.e. shared genetic pathways) [47–49]. LCV conceptually relies on a latent variable *L*, assumed to be the causal factor underlying the genetic correlation between both traits [47–49]. LCV estimates the genetic causality proportion (GCP). A higher absolute GCP value indicates more evidence of a causal association among a pair of genetically correlated phenotypes. In contrast, a GCP value of zero would imply that horizontal pleiotropy underlies the genetic correlation between the phenotypes. However, the LCV method will be biased towards the null (a GCP value of 0) if a bi-directional association exists between traits. An absolute value for GCP < 0.60 indicates only partial genetic causality. Multiple testing correction was applied using Benjamini–Hochberg’s FDR (FDR < 5%). We performed a phenome-wide hypothesis-free LCV analysis to identify traits causally associated with SA. Given the limitations of the LCV method (see “Discussion” section), we consider this a hypothesis-generating approach. These hypotheses should be tested in follow-up studies that include relevant Mendelian randomization analyses and a synthesis of the available literature on the association between SA and the trait of interest. In addition, as a sensitivity analysis, we performed two sample MR analyses for SHBG and vitamin D with SA (see Supplementary Methods).

## Results

### GWAS meta-analysis

**Figure 1.**
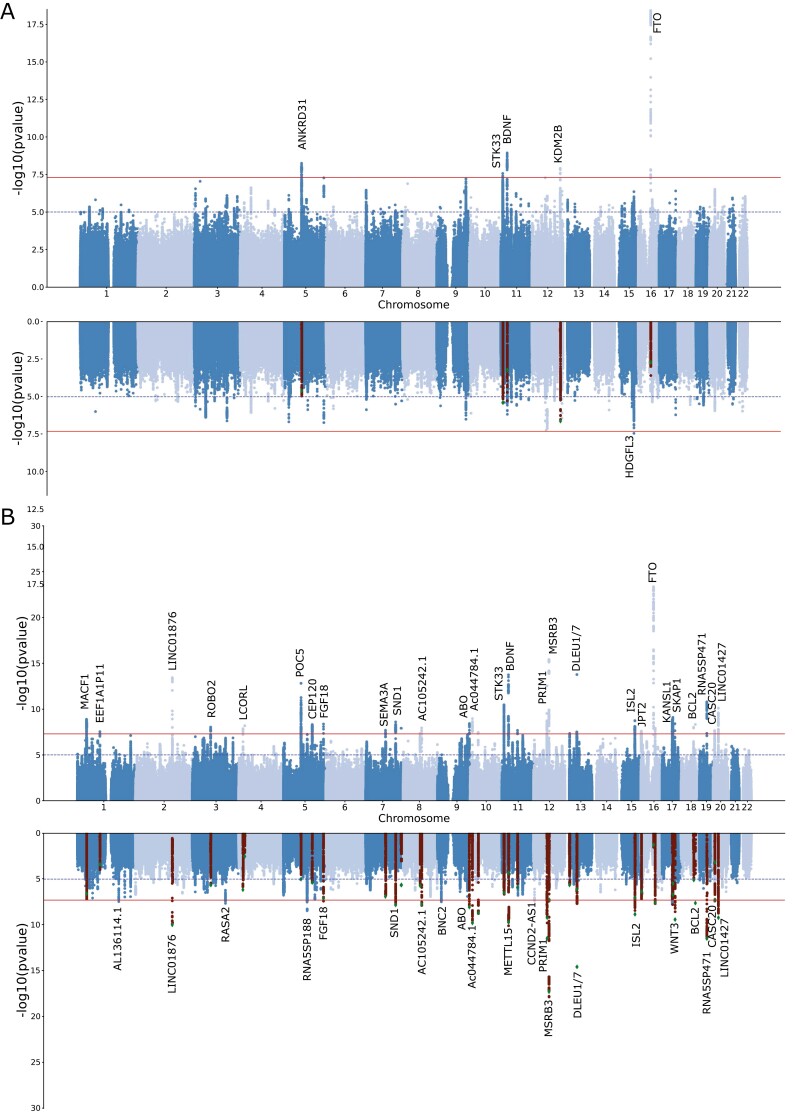
Discovery of genetic associations with sleep apnea (SA) risk. Miami plots depict the meta-analysis results for SA before and after adjusting for BMI using mtCOJO (A) or MTAG for SA plus snoring before and after adjusting for BMI using mtCOJO (B). Each dot represents a genetic variant. The *x*-axis represents the variant’s genomic position, and the *y*-axis depicts the significance of the association with SA. In the BMI-adjusted analyses, highlighted variants show the genome-wide hits of the unadjusted GWAS.

The prevalence of both SA and snoring showed some variation across the five cohorts included in this study (Table 1 and Supplementary Material). Nonetheless, all the genetic correlation estimates were high, albeit with large standard errors (Supplementary Table S1). Our meta-analysis identified five independent (LD *r*^2^ < 0.05) genome-wide significant (*p* < 5e−8) loci associated with SA (Figure 1, A). The signals spanned chromosomes 5, 11, 12, and 16 near genes *ANKRD31*, *STK33*, *BDNF*, *KDM2B*, and *PRIM1* (Supplementary Figure S2). The LD-score regression SNP-based heritability on the observed scale was 13% (S.E.  = 0.087%). Using a transformation that is more suitable for biobank structure [50], we estimate the heritability on the liability scale might range between 55% and 87% (based on an assumed population prevalence range of 9%–55%). LD-score regression intercept suggested most inflation (*λ*_GC_ = 1.21) was due to polygenic signal (intercept = 1.012, S.E. = 0.009) rather than population stratification. Upon adjusting for the effect of BMI using mtCOJO, one new genome-wide hit on chromosome 15, located near genes *HDGFL3*, *TM6SF1*, and *BNC1*, was identified (Supplementary Figure S2). A sensitivity analysis, including BMI as a covariate (see “Methods” section) also identified one single hit in chromosome 13. However, the evidence of association for all other loci was reduced below genome-wide significance upon adjustment for BMI (Figure 1, A). The genetic correlation between mtCOJO and covariate adjustment was 1.02 (S.E. = 0.024). Overall, the effects of most loci with suggestive evidence of association were consistent across the unadjusted, mtCOJO and covariate-adjusted meta analysis (Supplementary Figure S3). The only exception was the *FTO* locus that showed a statistically significant shrinking of effect upon BMI adjustment.

### MTAG

We used MTAG to boost statistical power and increase loci discovery by leveraging the genetic correlation between SA and snoring. This analysis had an effective sample size of 159 255 participants and identified 43 independent genome-wide significant loci associated with SA (Figure 1, B). The direction and effect sizes of the independent hits were highly consistent across the SA meta-analysis and the MTAG analysis with snoring (*R*^2^ > 0.95 Supplementary Figure S4). After adjusting for BMI using mtCOJO, 25 hits were genome-wide significant; most overlapped with the unadjusted results (Figure 1, B). We assessed whether previous genetic association studies of SA or related traits [26, 27, 51–55] align with our results and survive adjustment for BMI. We found some evidence of association for 5 of the 22 loci assessed. Two of the previously reported loci showed evidence of association after adjusting for BMI (Supplementary Table S2).

### Independent sample replication

We sought replication of our GWAS results in an independent sample (*N* = 1 477 352) from 23andMe. Notably, the 23andMe SA replication GWAS was adjusted for BMI (see “Methods” section). Overall, 10 of the independent variants identified by our analyses showed evidence of association beyond the genome-wide significance threshold (Supplementary Table S3) in the replication. After multiple testing corrections, three out of the five loci for SA meta-analysis were replicated. Furthermore, the variant that became significant after adjusting for BMI was also replicated. For the SA plus snoring MTAG, 30 out of 43 variants available in the 23andMe dataset were replicated. Finally, 22 out of 25 variants from the SA plus snoring MTAG adjusted for BMI analysis were also replicated. This higher replication rate was expected since the 23andMe GWAS had been adjusted for BMI (see “Discussion” section). Overall, 29 significant independent loci with evidence for replication were identified (Table 2). Furthermore, there was a large concordance in the direction and magnitude of effect sizes between our analyses and the 23andMe replication results (Supplementary Figure S5) and across cohorts (Supplementary Figures S6 and S7). Due to power, replication rates, and the interest in studying the etiology of SA beyond BMI effects, we focus below on the meta-analysis, the MTAG analysis, and the MTAG analysis adjusted for BMI.

**Table 2.** Independent hits associated with SA and replicated in 23andMe

SNP	CHR	BP	A1	A2	P_23&me	BETA^a^	SE^a^	P_META	Signal source
rs1537818	1	39647038	G	A	2.76E−05	−0.01755	0.004182	1.31E−09	MTAG
rs633715	1	177852580	T	C	4.60E−07	−0.02466	0.004898	3.49E−08	MTAG_BMIadj
rs72902175	2	157013035	T	C	9.30E−10	0.035999	0.005866	3.67E−14	MTAG
rs1403848	3	77609655	C	A	7.51E−05	−0.01569	0.003962	9.30E−09	MTAG
rs4076077	5	170863509	T	C	3.70E−06	−0.01797	0.003882	4.26E−09	MTAG
rs1428381	5	122693901	G	A	0.000369	0.015265	0.004283	4.83E−09	MTAG
rs2715039	7	84094964	C	A	4.61E−05	−0.01611	0.003952	2.04E−08	MTAG
rs7005777	8	78233600	T	G	5.18E−05	0.017513	0.00433	1.12E−08	MTAG
rs8176749	9	136131188	T	C	1.47E−05	−0.03212	0.007433	3.78E−09	MTAG
rs10756798	9	16739763	T	C	3.70E−09	−0.02425	0.004115	3.28E−08	MTAG_BMIadj
rs1444789	10	9064361	T	C	2.40E−13	−0.03701	0.005042	1.10E−09	MTAG
rs6265	11	27679916	T	C	1.12E−05	−0.02198	0.00501	1.79E−14	MTAG
rs1815739	11	66328095	T	C	1.19E−06	0.018979	0.003906	2.10E−08	MTAG
rs4923536	11	28422496	G	A	1.52E−10	0.025071	0.003915	7.51E−11	MTAG_BMIadj
rs28758996	12	121960480	G	A	0.00122	−0.01282	0.003963	1.21E−08	META
rs1389799	12	65824846	G	A	3.57E−25	0.04184	0.004032	1.38E−18	MTAG_BMIadj
rs4554968	12	4372609	G	A	0.000854	0.013381	0.004011	4.47E−08	MTAG_BMIadj
rs592333	13	51340315	G	A	9.04E−23	−0.03997	0.004068	1.69E−14	MTAG
rs11852496	15	83817559	T	C	3.17E−05	−0.01918	0.004605	1.71E−06	META
rs11634019	15	76634680	T	C	4.44E−10	0.027288	0.00438	1.84E−09	MTAG
rs11075985	16	53805207	C	A	1.13E−05	0.017161	0.003907	5.41E−20	META
rs8045335	16	60607116	G	A	1.41E−09	−0.02374	0.003922	1.24E−08	MTAG
rs9933881	16	1740691	T	C	3.68E−07	−0.03664	0.007183	2.54E−08	MTAG
rs12603115	17	46248994	T	C	3.95E−06	−0.01812	0.003926	8.14E−10	MTAG
rs227731	17	54773238	T	G	2.40E−11	−0.02603	0.003896	3.96E−09	MTAG
rs4987719	18	60960310	T	C	1.28E−08	0.061095	0.01068	4.72E−09	MTAG
rs35445111	19	32172047	G	A	2.62E−12	0.04751	0.006817	1.62E−11	MTAG
rs6113592	20	22229505	G	A	6.42E−07	0.019892	0.003998	7.82E−11	MTAG
rs6038517	20	6458205	G	A	0.000217	−0.01703	0.0046	2.19E−08	MTAG

GWAS and replication significant SNPs. Results are shown for variants with genome-wide evidence of association (*p* < 5e−8) in at least one of the main analyses, and evidence of replication in 23andMe. Abbreviations: META, sleep apnea meta-analysis; MTAG, sleep apnea plus snoring MTAG. MTAG_BMIadj, sleep apnea plus snoring MTAG adjusting for BMI using mtCOJO.

^a^Information based on the 23andMe GWAS.

### Gene-based tests and colocalization

The gene-based association analyses identified 22, 132, and 74 genes beyond the significance threshold (*p* < 2.07e−6) for the SA meta-analysis, the SA plus snoring MTAG, and the SA plus snoring MTAG adjusted for BMI respectively. As expected, many of these genes overlapped. Identified genes included *DLEU1, DLEU7, MSRB3*, *CTSF*, and *SCAPER* (Supplementary Figure S8 and Supplementary Tables S4–S6). Some of these genes were located within the same locus and in high LD. Thus, to identify genes linked to SA through potential changes in gene expression, we performed eQTL colocalization analyses for any of the genes mentioned above. Of the 151 genes with available eQTL data, only 18 showed strong evidence of eQTL colocalization with either SA, SA plus snoring or SA plus snoring adjusted for BMI (Supplementary Tables S7–S9).

### eQTL integration

We used S-MultiXcan to integrate our GWAS summary statistics with eQTL data and identify genes associated with SA through changes in predicted gene expression. These analyses identified 5 and 65 genes (Supplementary Table S10), for which evidence of association with SA meta-analysis or SA plus snoring MTAG reached statistical significance. These genes included *DLEU7, PRIM1, COPZ2, SKAP1, DNAJB7, ACTBP13*, and *ZBTB6,* among others. Although the results of S-MultiXcan partially overlapped those of the gene-based positional analysis, this approach identified 4 and 33 new genes that are likely associated with SA through changes in gene expression. Genes with convergent evidence through gene-based association and S-MultiXcan include *FTO, STK33, ETFA, SKAP1, MAPT, BAZ2A, DCAF16, MACF1, NSF, COPZ2, SP6, LACTB2, LRRC4*, and *HOXB3* among others (Supplementary Figure S8).

### Genetic correlations

**Figure 2.**
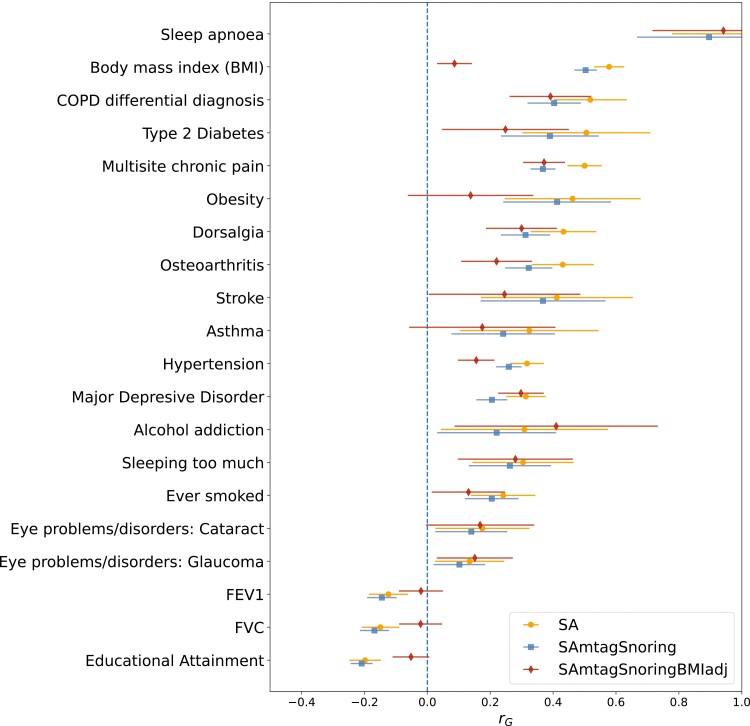
Sleep apnea (SA) is genetically correlated with psychiatric, behavioral, and cardiorespiratory traits. Forest plots showing genetic correlations calculated using CTG-VL [44] between SA meta-analysis, MTAG between SA and snoring (SAmtagSnoring) and MTAG between SA and snoring adjusted for BMI (SAmtagSnoringBMIadj). Markers depict the genetic correlation estimate (*r*_*g*_), whereas lines represent 95% confidence intervals derived from the *r*_*g*_ standard error. Not all traits with a significant association (FDR < 0.05) are shown. See the Supplementary Data for other traits.

Bivariate LD score regression was used to assess the genetic correlation between SA and other complex traits. The trait with the highest genetic correlation (*r*_*g*_ = 0.92) with SA was a SA GWAS performed on the UK-Biobank from a public GWAS repository (http://www.nealelab.is/uk-biobank/); this is essentially a subset of the UK-Biobank GWAS used in our meta-analysis. Other genetically correlated traits (*p*-value < .05) included respiratory diseases, type 2 diabetes, obesity, eye disorders, stroke, depression, alcohol addiction, smoking history, and musculoskeletal disorders such as arthritis and spondylosis, among others (Supplementary Tables S11–S13). The SA meta-analysis and the SA plus snoring analyses showed a highly concordant pattern of genetic correlations. While also showing overall agreement, the SA plus snoring adjusted for BMI results showed lower genetic correlations with BMI-related traits such as obesity, diabetes, and stroke (Figure 2).

### Polygenic risk scoring

**Figure 3.**
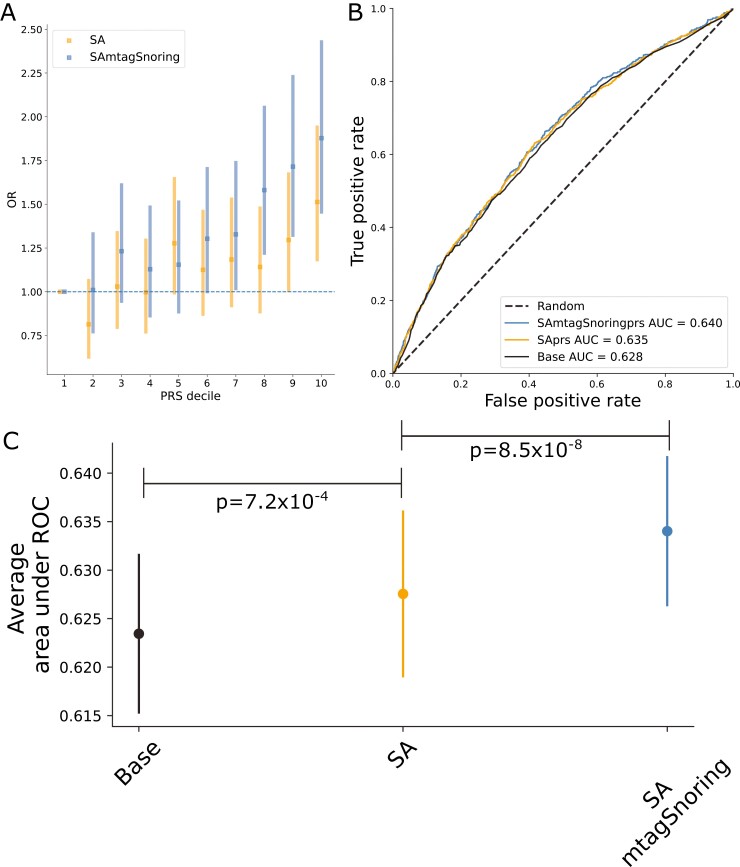
Sleep apnea (SA) polygenic prediction. (A) Plot showing the odds ratio (OR) per change in polygenic risk score (PRS) decile. Error bars depict the 95% confidence intervals. (B) Example of a receiver operating characteristic (ROC) curve derived from assessing the ability of logistic regression to predict SA using either a base model (covariates only) or the base model plus the PRS of interest. The higher the area under the curve, the higher the model’s predictive power. (C) Average area under ROC curve after 100 iterations of leave out validation randomly assigning training and testing subsamples. Error bars depict the standard deviation of the mean. Full results (100 ROC curves per model) are available in Supplementary Figure 9. Abbreviations: SA, sleep apnea meta-analysis; SAmtagSnoring, sleep apnea plus snoring MTAG.

PRS based on either of our results were significantly associated with SA in a leave one out polygenic prediction analysis. Odds ratios (OR) per standard deviation of PRS increased with the number of hits. For example, the meta-analysis-based PRS (SA_PRS_) showed an OR = 1.15 (1.08–1.21), whereas the PRS based on the SA plus snoring showed an OR = 1.21 (1.14–1.28). A similar pattern was observed for variance explained and significance (Table 3). These PRS were significantly associated with SA even after adjusting for BMI measures in the AGDS cohort (Table 3), suggesting that signals independent from BMI contribute to polygenic prediction. Participants in the highest PRS decile showed between 50% and 87% higher odds of reporting SA than participants in the lowest decile (Figure 3, A). Classifier models based on PRS showed a prediction ability higher than a random guess for the meta-analysis. The MTAG results showed an even higher predictive ability than the meta-analysis alone (Figure 3, B and C and Supplementary Figure S10).

**Table 3.** SA polygenic prediction

Model	OR (95% CI)	*P*	Nagelkerke *R*^2^ (%)	Variance explained (%)
SA_prs_	1.15 (1.08–1.21)	5.6e−06	0.4	0.45
SA_prs_ (adjusting for BMI)	1.09 (1.03–1.16)	4.2e−03	0.17	0.45
SAmtagSnoringprs	1.21 (1.14–1.28)	3.9e−10	0.77	0.87
SAmtagSnoringprs (adjusting for BMI)	1.14 (1.07–1.21)	2.4e−05	0.37	0.87

Results of PRS derived using our GWAS (leaving out the AGDS cohort) predicting sleep apnea in the AGDS sample with and without accounting for BMI measures in AGDS. Note significant prediction even after adjusting for BMI measures. Abbreviations: SAprs, sleep apnea meta-analysis; SAmtagSnoring, sleep apnea plus snoring MTAG.

### Predicting traits causally associated with SA

We used LCV to perform a hypothesis-free screening to assess whether the potential genetic overlap between SA and >400 traits and diseases can be explained by a causal relationship. To this end, we employed the results of the MTAG GWAS with snoring, given its increased statistical power. We did not identify any potential outcomes of SA. Nonetheless, we identified 103 potential causal determinants of SA (Supplementary Table S14). For instance, traits that purportedly increase the risk for SA, based on our analysis, included hypertension, asthma, lung cancer, obesity, having a period of mania, and hernia. Conversely, we found evidence for levels of vitamin D and sex hormone-binding globulin (SHBG) (from either a male- or female-only GWAS) to potentially reduce the risk for SA (Supplementary Figure S11). Two-sample Mendelian randomization sensitivity analyses did not identify evidence for a causal effect of vitamin D, but there was some evidence for a protective effect of SHBG quantile (females only) on SA (Supplementary Table S15). We repeated the LCV analysis approach using our BMI-adjusted summary statistics to test how many of these associations were explained by the large overlap with BMI. This identified 29 traits associated with SA (Supplementary Table S14; see “Discussion” section), six of which overlapped with the BMI-unadjusted-analysis mentioned above. These traits were medication taken for anxiety, angina pectoris, testosterone quantile (males), taking ibuprofen, walking for pleasure as physical activity, and depression diagnosed by a professional.

## Discussion

This study aimed at increasing our understanding of the genetic etiology of SA risk, an area that has stagnated due to the difficulty in achieving the required sample size for GWAS studies. Our SA GWAS meta-analysis combined data across five cohorts and identified five independent loci (Supplementary Figure S7). The evidence of association for most these loci decreased below statistical significance upon adjustment for BMI using both mtCOJO or including BMI as a covariate. Adjusting for BMI identified a new locus on chromosome 15 near *HDGFL3* when adjusting through mtCOJO and one on chromosome 13 near DLEU1 and DLEU7 when adjusting for BMI as a covariate. While this manuscript was under review, another study describing a GWAS for SASA in FinnGen and the UKB was published [51]. That study identified five genome-wide significant loci associated with SA and a clear, strong causal component of BMI. The strong influence of BMI is consistent with our observation of genome-wide hits showing weaker evidence of association upon adjustments for the effect of BMI [43]. We used MTAG to boost power and identify additional loci likely to confer SA risk by combining our SA meta-analysis with a snoring meta-analysis. We also identified several variants linked to SA over and above the effect of BMI and sought replication in an independent sample from 23andMe. The 23andMe GWAS adjusted for BMI, and we could replicate 29 loci associated with SA, suggesting our results are robust signals linked to other SA pathways.

We employed gene-based tests and identified several genes associated with SA, including *DLEU1, DLEU7 CTSF, MSRB3, FTO*, and *TRIM66*. The association with *FTO* is likely due to this loci’s strong effect on BMI and adiposity [56]. Loss-of-function of *MSRB3*, which encodes a methionine sulfoxide reductase, has been associated with human deafness. This finding is consistent with reported associations between hearing impairment and SA [57]. *CTSF* has been linked to the airway wall area (Pi10) as measured quantitatively using CT chest images [58]. That is consistent with the fact that small airway dimensions have been linked to SA measures in a COPD comorbid sample [59] and that obesity is believed to increase SA risk increasing the fat levels of upper airway structures and the compression of airway walls [60]. *DLEU1* and *DLEU7* are both located within a region associated with leukemia. While *DLEU7* is a protein-coding gene, *DLEU1* was recently discovered to be part of a bigger gene, *BCMS,* that has a potential tumor-suppressing function [61]. Although this locus has been linked to snoring [33], its role in the pathogenicity for SA remains to be clarified.

Genes with evidence from positional gene mapping and gene-expression integration included *SKAP1, MAPT, STK33*, and *ETFA,* among others. *SKAP1, STK33*, and *MAPT* are genes related to the MAPK signaling pathway. *MAPT* is genetically and neuropathologically associated with neurodegenerative disorders, including Alzheimer’s disease and frontotemporal dementia [62]. Furthermore, *ETFA* expression has been observed to change in an Alzheimer’s disease mouse model in response to aducanumab, an amyloid beta antibody [63]. There is a known link between SA and Alzheimer’s disease [64]. Recent studies with mouse models suggest that intermittent hypoxia induces cholinergic forebrain degeneration [65]. Furthermore, other observations suggest SA severity might be linked to increased amyloid-beta plaques [66]. Although informative, these studies still lack the ability to distinguish whether a true causal association underlies SA and Alzheimer’s disease in humans. Our results should enable the exploration of this question by enabling causal inference studies using instrumental variable analysis.

We did not replicate previously reported candidate gene associations such as *TNFA*, *APOE*, *PTGER3*, and *LPAR124*. This could be explained by differences between our analysis and those identifying the candidate genes. For example, the *LPAR1* association was observed in participants of African ancestry [67]. Nonetheless, studies assessing the support for candidate gene associations using GWAS have found poor consistency [68]. Our results suggest a similar trend for candidate gene studies of SA. Our study should be powered to detect previously reported candidate-gene effect sizes; for instance, polymorphisms within *TNFA* were reported to show an odds ratio of 2.01 for SA [69]. Future studies should systematically evaluate candidate gene studies and GWAS concordance in SA, an objective that was outside the scope of the current study.

As a proof-of-principle of the utility of having well-powered GWAS summary statistics, we performed a hypothesis-free inference of causal associations between >400 traits and our SA MTAG. Consistent with previous findings [51], our approach inferred obesity to likely increase the risk for SA. Similar results were found for asthma, lung cancer, hernia, hypertension, a period of mania, and stroke. Conversely, we found that SHBG levels derived from male-only, female-only, and combined-sex GWAS decreased the risk for SA. A similar finding was observed for endogenous testosterone levels derived from a male-only GWAS. This is consistent with observations of SHBG and testosterone levels negatively correlating with SA severity [70]. However, continuous positive airway pressure therapy does not seem to reverse these abnormal changes [71, 72], which would be consistent with the direction of causality predicted through LCV (from hormone level to phenotype). LCV also identified vitamin D levels as causal determinants of SA risk. That is consistent with reports linking vitamin D with SA [73]. Nonetheless, it is also possible that this result is explained by BMI. Given that vitamin D levels increase with sun exposure [74], and exposure increases with physical activity, the well-documented inverse relationship between obesity (or BMI) and vitamin D concentrations might better explain the observed association [75, 76]. The extent to which hypertension, hernia, and stroke are associated with SA above and beyond obesity as a shared causal component was unclear. We tested this by performing our causal analyses using BMI-adjusted summary statistics. Our results suggest most of these associations are potentially mediated through BMI, as these associations were no longer significant after adjusting for BMI. Interestingly, a lifetime diagnosis of depression was consistently associated with an increased risk for SA, even after adjusting for BMI. Overall, our LCV analysis identified a set of testable hypotheses, which can be further explored through multivariable MR analyses contrasting the observational associations with SA, and genetically derived effect sizes for SA and BMI. We performed MR as sensitivity analyses for LCV and found further evidence for a protective effect of SHBG. The SHBG GWAS for which evidence was identified was performed within females only, thus, we do not anticipate these results to be explained by a bias arising from sex differences on SHBG. On the contrary, SHBG levels may be one of the factors involved in the differential prevalence of SA between males and females. Two sample MR studies should be performed on GWAS without sample overlap and including a range of sensitivity checks to rule out heterogeneity and pleiotropy. Such comprehensive analyses are outside the scope of this study.

This study was performed using cohorts of European ancestry. Thus, generalizations and comparisons with other ancestry groups should be performed with caution. In order to maximize sample size, we included cohorts with different definitions of SA, including ICD codes and patient-reported diagnosis. The effect of these definitions is not negligible, as SA prevalence displayed marked differences (i.e. up to sixfold) between cohorts. The AGDS and CLSA cohorts use a single question that assesses whether a participant stops breathing during sleep. This item could also capture cardiopulmonary diseases. Furthermore, although ICD-10 codes may be considered a gold standard for ascertaining cases in GWAS studies, there are reports of low specificity [77] when identifying cases for sleep disorders. To avoid contamination from potentially undiagnosed cases in the control group, we have strived to remove participants that report loud snoring from the control set. While combining multiple sources for phenotype definition is warranted to achieve the required sample sizes for GWAS, minimal phenotyping might introduce heterogeneity. Future studies should explore using novel advances in natural language processing [78] of electronic health records to increase the accuracy of biobank-based phenotyping and compare the accuracy and genetic concordance of the different phenotyping approaches used here. We found the combined effect of the SNPs in our meta-analysis to explain ~13% of the variance of SA on the observed scale. Estimating heritability on the liability scale is challenging given (i) the wide range of reported prevalence in the population (9%–55%) and (ii) the fact that the current adjustments for transforming between the observed and liability scale assume an overrepresentation rather than an underrepresentation of cases. To avoid this issue, we have used a recently developed model to estimate liability scale heritability on samples with these characteristics [50].

Our results for cross-cohort pairwise genetic correlations suggested that despite using different phenotype ascertainment methods, the underlying genetics represent a common trait. Nonetheless, this analysis suffered from reduced power, and the large standard errors do not allow us to rule out heterogeneity across cohorts. Ideally, any SA study would ascertain cases employing a robust measure such as the apnea–hypopnea index or oxygen saturation; GWAS of complex traits require enormous sample sizes, making such approach challenging. Although MTAG has proven successful in boosting the discovery of loci associations, even in the presence of known or unknown sample overlap [29], combining traits with extreme power differences might inflate signals related to the most powered phenotype [29]. The genetic correlation between snoring and our MTAG analysis was higher than that with the SA meta analysis alone. This increase in correlation is not unexpected as MTAG can only boost power for the shared genetic component between the traits included in the multivariate analysis. However, the addition of snoring through MTAG nearly doubled the effective sample size of the SA GWAS (*N*_eff_ increase from ~90 000 to ~159 000), which resulted in identifying several novel loci with evidence of replication even after adjusting for BMI and the generation of summary statistics of utility for analyses such as MR and PRS.

In our study, adjusting for BMI seemed to affect the pattern of genetic correlations, particularly decreasing the correlations with BMI and related traits such as stroke and obesity. Replication of the SA plus snoring adjusted for BMI results was higher than in the other analyses. This result is expected for two reasons: First, it benefited from the increased power of combining GWAS for apnea and snoring through MTAG and adjusted for BMI using mtCOJO. Second, the GWAS performed by 23andMe included BMI as a covariate. As such, it resembles a phenotype in line with those for which the SA plus snoring adjusted for BMI is boosting power. Finally, some limitations of the approach used for causal inference need to be acknowledged. LCV is still dependent on the power of the original GWAS for both traits. Traits with a potential causal association with SA might not have been included in the tested traits. Finally, this method assumes no bi-directional causality and will likely be biased towards the null in such cases. Thus, a null finding in our study does not reflect a lack of association, especially if bidirectional relationships are suspected.

In summary, we performed a GWAS meta-analysis of SA across five European-ancestry cohorts and identified five independent genome-wide significant loci. Conditional analyses suggested a large contribution of BMI to SA; most of the discovered genome-wide hits in the meta-analysis were explained by BMI. After adjusting for BMI, the meta-analysis identified one genome-wide significant locus. MTAG of SA with snoring identified 43 independent hits and 23 after conditioning on BMI. Overall, 29 independent significant hits were replicated in an independent SA GWAS from 23andMe. All analyses showed a significant polygenic prediction of SA in a leave-one-out PRS analysis. Our results largely confirm the previously observed overlap with BMI and highlight genetic overlap with traits such as stroke, asthma, hypertension, glaucoma, and cataracts. We further found evidence of a potential causal role of SHBG and vitamin D levels in decreasing the risk for SA. If confirmed by multivariable MR and interventional studies, new treatments based on modifying these risk factors might be used for SA treatment or early intervention. This general hypothesis-free framework can be used to generate testable hypotheses of risk factors for complex traits [49]. Also, the associations identified here can be used as instrumental variables in targeted MR studies aiming at understanding the relationship between SA and hypothesized causally related traits. Identifying robust loci associated with SA is an important step towards a deeper biological understanding, which can translate into novel treatments and risk assessment strategies.

## Supplementary Material

zsac308_suppl_Supplementary_Material

## Data Availability

The full GWAS summary statistics for this study will be available through the NHGRI-EBI GWAS Catalogue (https://www.ebi.ac.uk/gwas/downloads/summary-statistics). Data are available from the Canadian Longitudinal Study on Aging (www.clsa-elcv.ca) for researchers who meet the criteria for access to de-identified CLSA data. UK Biobank and FinnGen data are also accessible through their respective application procedures. The full GWAS summary statistics for the 23andMe discovery data set can be made available through 23andMe to qualified researchers under an agreement with 23andMe that protects the privacy of the 23andMe participants. Please visit research.23andme.com/collaborate/#publication for more information and to apply to access the data.
